# Individuals with depressive tendencies experience difficulty in forgetting negative material: two mechanisms revealed by ERP data in the directed forgetting paradigm

**DOI:** 10.1038/s41598-018-19570-0

**Published:** 2018-01-18

**Authors:** Hui Xie, Donghong Jiang, Dandan Zhang

**Affiliations:** 1 Institute of Affective and Social Neuroscience, Shenzhen University, Shenzhen, 518060 China; 2 Shenzhen Key Laboratory of Affective and Social Cognitive Science, Shenzhen University, Shenzhen, 518060 China

## Abstract

Although previous studies have shown that individuals with depressive tendencies have deficits in forgetting negative material, the detailed underlying neural mechanisms have not been elucidated. This study examined the intentional forgetting of negative and neutral material in individuals with depressive tendencies in two phases. In the study phase, the participants performed a directed forgetting task, where a total of 320 words were presented to them, each followed by an instructive cue to forget or remember the previously presented word. Subsequently, in the memory recognition test phase, the participants completed the “old or new discrimination task”. The results indicated that individuals with depressive tendencies had difficulties suppressing the memory encoding of negative words, while the suppression of memory encoding of neutral words was relatively intact. Moreover, individuals with depressive tendencies displayed enhanced word-evoked P2 and late positive potential for negative items, as well as enhanced cue-evoked P1 and N2 for the negative items that were required to be forgotten, as compared to individuals without depressive tendencies. Based on these results, we propose two mechanisms that may contribute to the failure of forgetting negative material in mild depression: (1) inefficient memory suppression and early selective attention, and (2) excessive preliminary processing.

## Introduction

According to Beck’s cognitive model of depression, depressed individuals exhibit excessive negative biases in many aspects of cognitive processes, such as attention and memory, as compared to healthy controls^1,2^. This negative bias plays a critical role in the etiology as well as the maintenance of depressive symptoms (e.g. the ruminative thinking style)^3–6^. The majority of previous studies focused on the biased processing of emotional information in the aspects of perception, attention, memory and interpretation, neglecting the functions of inhibition and cognitive control in the depressed population^2,7^. Recent studies have demonstrated that depressed individuals are less successful in excluding unwanted thoughts and memories from awareness due to inhibitory deficits in the cognitive control system^8–10^. This impaired inhibitory function may consequently hinder recovery from depressive symptoms, and induce a vicious circle of a mood-congruent state and a persistent low mood^7,11^. Therefore, it is important to investigate the intentional forgetting and the associated neural mechanisms in the depressed population, which may broaden our understanding of the pathology of this disorder and aid in the development of more efficient treatments.

As reviewed by Anderson & Hanslmayr^12^, memory inhibition or suppression may occur during the stages of memory encoding and memory retrieval. The intentional forgetting at the stage of memory encoding can efficiently limit and disrupt the consolidation and further reflection of unwanted memories, which is the focus of this study. Memory inhibition during encoding is usually investigated using the directed forgetting (DF) paradigm^13^, which consists of two phases. In the study phase, participants are presented with a series of items, some of which are “to be remembered” (TBR) and others that are “to be forgotten” (TBF). When the “remember” cue is given, participants are required to remember or consolidate the previously presented item. On the contrary, when the “forget” cue is given, they should try their best to forget the previously presented item. In the memory recognition test phase, participants complete the “old or new discrimination task”, where the previously studied words are mixed with new words. The DF effect is defined as a higher recognition rate for TBR than for TBF items in the phase of memory recognition test^12^. There are two methods to perform the DF task: the item-method, and the list-method^12,14^. In the item-method, the “remember/forget” cue is presented after each item, whereas in the list-method, the cue is presented after a list of items. Both methods involve activation of the frontal regions, but their cognitive and neural mechanisms are quite different^12^. This study employed the item-method paradigm, since it provides an insight into how individuals with depressive tendencies control new, incoming information.

Previous DF studies have shown that, while the medial-temporal lobe, and especially the hippocampus, is significantly activated by the “remember” instruction, the prefrontal and parietal regions are activated by the “forget” instruction^15–18^. In particular, intentional forgetting recruits more executive control resources at the frontal lobe than incidental forgetting^18^. Furthermore, some studies demonstrated a negative correlation between activity in the dorsolateral prefrontal cortex and the hippocampus during successful forgetting^17^. Thus, the psychological mechanism of the DF task has been proposed as follows: items are tentatively kept in the working memory until the “remember/forget” instruction requires participants to selectively rehearse and elaborately encode the TBR items or to disrupt the encoding and suppress the consolidation of the TBF items using the inhibitory control mechanism^15–24^. In addition to *selective rehearsal* and *inhibition*, another three mechanisms have been proposed for the item-method DF, namely, *set segregation* (TBR and TBF items are organized into two groups so that the processing of the items of each group does not interfere with the other^25^), *attentional withdrawal* (attention is withdrawn from the task-irrelevant TBF items so that their representations are removed from the working memory^26^), and *cognitive load* (forgetting is more effective and easier when cognitive resources are allocated to the rehearsal of TBR items or exhausted by other processes^27,28^).

Behavioral studies have revealed that individuals with high levels of depression exhibit deficits in directed forgetting; depressed patients^29^ and individuals with depressive tendencies^30^ recognized more TBF words than nondepressed individuals, more specifically, they exhibit reduced forgetting of negative, compared to neutral, or positive TBF words^31,32^. However, to date, the neural mechanisms underlying the memory control deficits in the depressed population have only been explored in two empirical studies. In one study, Yang *et al*.^9^ found that depressed patients could not forget negative material and displayed stronger activation in their frontal and parietal regions (associated with inhibitory control function) when they attempted to forget negative TBF items. This abnormal activation indicated that even though more inhibitory control resources were recruited to terminate the rehearsal of negative TBF items, the intent failed due to excessive negative bias. Therefore, it was postulated that depressed patients are inefficient in forgetting negative material^9^. In the other study, Berman *et al*.^8^ demonstrated that patients with major depressive disorder have difficulty in forgetting negative words, using a DF procedure. They observed that when participants expelled negative information from their short-term memory, both major depressive patients and healthy controls robustly activated a network (including the inferior frontal gyrus) that has been implicated in memory selection and interference resolution; there was no difference in the magnitude of activation of the two groups. However, while the control group displayed focal activation in the left inferior frontal gyrus, the major depressive patients exhibited more diffuse activation (i.e., a larger spatial variability) in this region; this spatial variability was associated with the patients' greater behavioral variance in reaction times.

Although the two fMRI studies^8,9^ demonstrated that depressed patients have abnormal brain activation when they attempt to forget negative material, the detailed neural mechanisms associated with their failure in the DF task is still unclear. As introduced above, there are two procedures in the study phase of the DF paradigm: the participants first preliminarily encode the presented item and then implement the “remember/forget” cue to remember or forget the encoded item. However, when using the fMRI technique, it is difficult to discriminate these two quick procedures due to its low temporal resolution. In this context, the authors of the present study considered that the event-related potential (ERP) is a more suitable tool to separately investigate the neural deficits in the two procedures. Moreover, another catalyst for the execution of this study was to shed light on the previous DF studies in healthy subjects, which presented contrasting results associated with the memories of emotional vs. neutral material^20,23,33,34^, that may be due to individual differences in these studies. To examine the impact of individual differences on the performance in the DF task, this study included measures of individual differences in depressive tendency, trait anxiety, ruminative tendency, and approach/avoidance motivational system. Subsequently, the relations between individual differences and behavioral/ERP indexes were explored.

In accordance with previous DF studies in healthy subjects, the present work focused on three ERP components. The first one is the middle frontal N2, which reflects cognitive control and is usually observed in motor and memory inhibition tasks^35–38^. It has been established that the “forget” instructive cue evokes larger N2 amplitudes (more negative-going potentials) than the “remember” cue^19,21,23,34^. In some DF studies, the N2 (approximately 200 to 300 ms after cue onset) was also referred to as “the frontal positivity”, since this negative-going component actually had positive amplitude^19,21,34^. The second component of interest is the parietal P3. This component usually displays larger amplitudes following the “remember” cue compared to the “forget” cue, reflecting selective rehearsal of the TBR items^19–23,33,34,39,40^. In addition to the cue-evoked N2 and P3, the word-evoked ERPs are also examined. Specifically, previous studies indicated that the late positive potential (LPP) displays larger amplitudes for negative items than for neutral items, indicating more elaborate processing of negative material in healthy individuals^20,23,33^. It should be noted that, both P3 and LPP refer to large and slow positive potentials, which peak at the parietal electrodes later than 300 ms post-stimulus. In this study, the term “P3” is used to refer to the relatively earlier positive component (about 300 ms) with a sharper peak, and the term “LPP” to refer to the slower positive wave (after 500 ms) with a blunter peak^41,42^. In addition to the three ERP components that have been often mentioned in the DF paradigm, the present study investigated early components such as the occipital P1 and the frontal P2, which are well-known attention-related components^43,44^, but have not been explored in previous DF studies. Although both P1 and P2 are usually used as indexes of selective attention, P1 is more sensitive to stimulus features (feature-based attention^45^) and arousal levels^46^, while the anterior P2 is often associated with the motivational salience of a stimulus as determined by either task-relevant features or novelty^47–51^.

This study compared the behavioral and ERP data of individuals with and without depressive tendencies. It is hypothesized that individuals with depressive tendencies experience difficulty in forgetting negative material, which is due to deficits/dysfunctions in both procedures during the study phase of the DF task, i.e., the item encoding, and the implementation of the “remember/forget” cue. Accordingly, it is expected on the behavioral level that subjects with depressive tendencies will have a higher recognition rate for TBF negative words than nondepressed subjects. To examine the deficits during the encoding procedure, the word-evoked ERP components of occipital P1, frontal P2 and parietal LPP were investigated. While to examine the deficits in the implementation of the “remember/forget” cue, the cue-evoked ERP components of occipital P1, frontal N2 and parietal P3 were investigated. It was hypothesized that subjects with depressive tendencies would excessively encode and rehearse negative items compared with nondepressed subjects. Considering that the amplitude of word-evoked LPP is associated with elaborate encoding processing^20,23,33^, it was predicted that negative words may elicit larger word-evoked LPP in subjects with depressive tendencies than in nondepressed subjects. Furthermore, considering that the amplitude of cue-evoked P3 is associated with selective rehearsal^19–23,33,34,39,40^, it was predicted that negative TBR words may elicit larger cue-evoked P3 in subjects with depressive tendencies compared with nondepressed subjects. Moreover, since Yang *et al*.^9^ demonstrated that depressed patients presented stronger activations in the neutral networks associated with inhibitory control function, it was hypothesized that inhibitory control resources might be inefficiently recruited in subjects with depressive tendencies when they are required to forget negative words. Accordingly, it was expected that negative words may elicit larger cue-evoked N2 amplitudes for TBF items in subjects with depressive tendencies compared to nondepressed subjects. Furthermore, this study examined the potential influence of individual differences (measured by several self-reported scales) on the performance of DF. According to previous studies using emotional material, the performance of DF might be affected by individual differences in depression^8,29,30^, rumination^9,31^, trait anxiety^30,32^, and the behavioral activation/inhibition system^52^. Consequently, this study included measures of individual differences in depression, ruminative tendencies, trait anxiety, and the activation/inhibition system (see detailed information in Methods).

## Methods

### Participants

Considering that psychiatric medications may affect behavioral and/or ERP results^53,54^, this study examined the impaired directed forgetting in individuals with depressive tendencies rather than in depressed patients. Furthermore, because previous studies have demonstrated a correlation between anxiety and deficits of directed forgetting^32,55,56^, this study only recruited participants with a moderate level of trait anxiety, i.e., the directed forgetting was compared between the individuals with and without depressive tendencies, and both the groups had a moderate level of anxiety.

In a mental health screening of Shenzhen University, all the freshman students (n = 6000) completed the Beck Depression Inventory Second Edition (BDI-II^57^) and the Trait form of Spielberger's State-Trait Anxiety Inventory (STAI-T^58,59^). In this sample, students with STAI-T scores within the middle 50% (from 25% to 75%) of the distribution were considered as subjects with a moderate level of trait anxiety^60,61^. Among the individuals with a moderate level of trait anxiety, students with BDI-II scores ≤ 13 were labeled as individuals without depressive tendencies, whereas students with BDI-II scores > 13 were labeled as individuals with depressive tendencies. According to Beck *et al*.^57^, a BDI-II score ≤ 13 indicates minimal depression, and a BDI-II score > 13 indicates mild (14–19), moderate (20–28), or severe depression (29–63). Among the students who met all these criteria, we randomly invited 60 individuals to participate the current study (30 individuals with and 30 individuals without depressive tendencies). As shown in Table 1, no significant difference was found between the two groups with respect to age, sex, handedness and STAI-T scores.

**Table 1:** Demographic data of the two groups. Descriptive data are presented as mean ± standard error.

Characteristics	Without depressive tendencies (n = 30)	With depressive tendencies (n = 30)	Statistics
Mean age, y	18.3 ± 0.1	18.5 ± 0.1	*t*(58) = −1.40, *p* = 0.167
Sex, male/female	16/14	15/15	
Handedness, right/left	30/0	30/0	
BDI-II	4.0 ± 0.4	19.8 ± 1.1	*t*(58) = −13.2, *p* < 0.001
STAI-T	41.9 ± 1.1	44.8 ± 1.1	*t*(58) = −1.84, *p* = 0.072
RRS	36.2 ± 1.1	56.0 ± 2.0	*t*(58) = −8.76, *p* < 0.001
BIS	19.7 ± 0.6	24.0 ± 0.7	*t*(58) = −4.48, *p* < 0.001
BAS	42.0 ± 0.8	42.3 ± 1.3	*t*(58) = −1.96, *p* = 0.846

BDI-II, Beck Depression Inventory (Second Edition).

STAI-T, the Trait form of Spielberger's State-Trait Anxiety Inventory.

RRS, the Rumination Response Scale.

BIS/BAS, the Behavioral Inhibition System and Behavioral Activation System Scale.

Exclusion criteria for both groups were 1) any Axis I and II disorders according to the Diagnostic and Statistical Manual (DSM-IV)^62^; 2) seizure disorder; 3) history of head injury with possible neurological sequelae, and 4) substance abuse or dependence in the past six months.

Participants were told about the equipment used in the experiment and their tasks. Written informed consent was obtained prior to the experiment. The experimental protocol was approved by the Ethics Committee of Shenzhen University and this study was performed strictly in accordance with the approved guidelines.

### Self-reported measures

The BDI-II is a widely used self-reported measure of depressive symptoms^57^. It consists of 21 items that assess the level of depressive symptoms in the past two weeks. The BDI-II scores from 0 to 63; a high score indicates a high level of depressive tendency.

The STAI-T is developed to evaluate a relatively enduring tendency of anxiety^58,59^. It contains 20 items and scores from 20 to 80; a high score indicates a high level of trait anxiety.

The Ruminative Response Scale (RRS)^63–65^ is used to assess how participants tend to respond to sad feelings and symptoms of dysphoria. It contains 22 items and scores from 22 to 88; a high score indicates a high level of ruminative tendency.

The Behavioral Inhibition System and Behavioral Activation System Scale (BIS/BAS) is designed to assess individual differences in the sensitivity of two motivational systems, i.e., a behavioral approach system and a behavioral avoidance system^66^. The BIS sub-scale contains 7 items (scores from 7 to 28) and the BAS contains 13 items (scores from 13 to 52). A high score on BIS or BAS indicates a larger tendency to regulate aversive or appetitive motives so as to move away from unpleasant, or to move toward desired, events and stimuli.

### Experimental design and stimuli

A design with two (instruction: remember *vs*. forget) × two (valence of material: neutral *vs*. negative) × two (group: with depressive tendencies *vs*. without depressive tendencies) factors was used in this study.

This study used 640 words (320 negative and 320 neutral ones) from Chinese Affective Words System^67^, with equal number of words between adjectives and nouns. Each word consisted of two Chinese characters. The material was assessed for its familiarity, valence and arousal on a 9-point scale with a large sample of Chinese participants in a previous survey. The negative and neutral words had a median level of arousal (negative = 5.00 ± 0.80, neutral = 4.43 ± 0.64; *t*(638) = 10.0, *p* < 0.001), and the two categories differed significantly in valence (negative = 3.52 ± 0.75, neutral = 5.76 ± 0.50; *t*(638) = −44.4, *p* < 0.001). No difference was found in the familiarity between negative and neutral words (negative = 5.32 ± 0.59, neutral = 5.33 ± 0.58; *t*(638) = −0.17, *p* = 0.868).

Considering that the DF paradigm consisted of two phases (study and recognition test)^13^, the 640 words were randomly divided into two subsets, with equal number of words between neutral and negative conditions and between nouns and adjectives. One subset (320 words) was used in the study phase while the whole 640 words were used in the recognition test (i.e., 320 new and 320 old words). The valence (negative: *t*(318) = −0.96, *p* = 0.336; neutral: *t*(318) = −0.85, *p* = 0.397), arousal (negative: *t*(318) = 0.39, *p* = 0.694; neutral: *t*(318) = 0.32, *p* = 0.748), and familiarity (negative: *t*(318) = 1.28, *p* = 0.201; neutral: *t*(318) = −0.20, *p* = 0.840) of negative and neutral words were counterbalanced between the two subsets. Furthermore, the valence (negative: *t*(158) = −1.36, *p* = 0.176; neutral: *t*(158) = −1.25, *p* = 0.213), arousal (negative: *t*(158) = 1.29, *p* = 0.199; neutral: *t*(158) = 1.14, *p* = 0.255), and familiarity (negative: *t*(158) = −0.56, *p* = 0.579; neutral: *t*(158) = −0.55, *p* = 0.580) of negative and neutral words were counterbalanced between the “to be remembered” and “to be forgotten” conditions. The allocation of the words to each experimental condition was not changed during the experiment, while the presentation order of words within one condition was randomized across subjects.

### Procedure

Before the DF task, participants were required to complete the four questionnaires (BDI-II, STAI-T, RRS and BIS/BAS).

**Figure 1:**
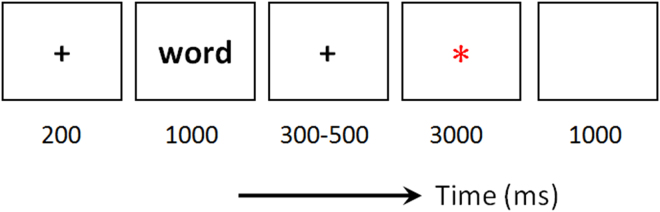
Illustration of one directed forgetting trial. Participants were required to forget or remember the previously presented word in response to the red or green asterisk. The assignment of colors to “forget” and “remember” tasks was counterbalanced across participants.

In the study phase, the 320 words were sequentially presented and each word appeared only once. The phase was comprised of four blocks (80 words in each block), separated by self-paced rest periods. As shown in Fig. 1, the word was presented for 1 sec and the instructive cue was presented for 3 sec. The cue was a green or a red asterisk, requiring participants to forget or remember the previously presented word (forget vs. remember = 50% vs. 50%). The assignment of colors to “forget” and “remember” instructions was counterbalanced across participants.

In the phase of memory recognition test, the old/new discrimination task was performed. The 320 studied words were mixed with another 320 new words. The words were sequentially presented and each word appeared only once. Participants were required to discriminate as quickly and as accurately as possible whether the word was presented in the study phase, irrespective of the “forget/remember” instructions. They responded by pressing the “F” or “J” button on a computer keyboard with their left or right index finger. The assignment of keys to old and new words was counterbalanced across participants. The inter-trial interval was 2000 ms. The phase was comprised of four blocks (160 words in each block), separated by self-paced rest periods.

A task-irrelevant task (mathematical ability test, 10 min) was filled between the study phase and the recognition phase (see also other studies^17,20,23,34^).

### EEG recording and analysis

Brain electrical activity was recorded referentially against left mastoid and off-line re-referenced to the average of the left and right mastoids, using a 64-channel amplifier with a sampling frequency of 250 Hz (Brain Products, Gilching, Germany). Electroencephalography (EEG) data were recorded during the study phase, with electrode impedances kept below 5 kΩ. Ocular artifacts were removed from EEGs using a regression procedure implemented in NeuroScan software (Scan 4.3). An on-line analogue filter (0.01–100 Hz) and a notch filter (50 Hz) continuously worked during the recording.

The recorded EEG data were off-line filtered (0.01–30 Hz) and segmented beginning 200 ms prior to the onset of stimulus and lasting for 1200 ms. Trials contaminated with large artifacts (peak-to-peak deflection exceeded ±200 μV) were excluded from further analyses. As a result, 74 ± 6.3 trials (mean ± std), 76 ± 4.9 trials, 75 ± 5.0 trials, and 74 ± 6.1 trials per subject were left in the group without depressive tendencies for the “remember-negative”, “remember-neutral”, “forget-negative”, and “forget-neutral” conditions. Meanwhile, 74 ± 7.1 trials, 73 ± 7.6 trials, 74 ± 6.2 trials, and 75 ± 5.8 trials per subject were left in the group with depressive tendencies for the four conditions (there were a total of 80 trials in each condition). All epochs were baseline-corrected with respect to the mean voltage over the 200 ms preceding the onset of stimulus, followed by averaging in association with experimental conditions.

This study focused on the ERPs elicited by words and instructive cues. Although ERP data were recorded from all the 64 electrode sites, we analyzed only the sites at which the component of interest was large and the waveform showed a representative pattern. Time windows for mean amplitude calculation were centered at the peak latencies of ERP components in grand-mean waveforms, with a shorter window length for early components and a longer length for late components.

For the word-evoked ERP, we analyzed average amplitudes of occipital P1, frontal P2 and parietal LPP components. The P1 amplitude was calculated as the average amplitude at the electrode sites of O1 and O2 between 80 to 120 ms after the onset of words. The P2 amplitude was calculated as the average amplitude at the electrode sites of F1, F2 and Fz between 220 to 270 ms post stimulus. The LPP amplitude was calculated as the average amplitude at the electrode sites of P1, P2 and Pz between 500 to 900 ms post stimulus.

For the cue-evoked ERP, we analyzed average amplitudes of occipital P1, frontal N2 and parietal P3 components. In particular, the P1 amplitude was calculated as the average amplitude at the electrode sites of O1 and O2 between 115 to 155 ms after the onset of cues. The N2 amplitude was calculated as the average amplitude at the electrode sites of F1, F2 and Fz between 240 to 290 ms post stimulus. The P3 amplitude was calculated as the average amplitude at the electrode sites of CP1, CP2 and CPz between 260 to 460 ms post stimulus.

It should be stated that the analysis of the word-evoked P1 and P2, and cue-evoked P1 was a post-hoc decision based on our data, because these components have not been reported in previous DF studies.

### Statistics

Descriptive data were presented as mean ± standard error, unless otherwise mentioned. The significance level was set at 0.05.

Repeated-measures ANOVA was performed on behavioral and ERP measurements, with instruction (remember *vs*. forget) and the emotion category of words (negative *vs*. neutral) as within-subject factors, and group (individuals with depressive tendencies *vs*. individuals without depressive tendencies) as the between-subject factor. Significant interactions were analyzed using simple effects model. Bonferroni correction was used for multiple comparisons.

Two-tailed Pearson’s *r* correlation was performed between the five self-reported measures and behavioral/ERP data. Correction for multiple comparisons was based on Holm’s stepwise method. To test the independency of one correlation, partial correlation was used to test correlation between a given self-reported measure and behavioral/ERP data while controlling for the other four self-reported measures.

## Results

For the sake of brevity, this section only reports the most important results. Please refer to the supplementary material for the other significant findings.

### Behavioral data

#### Recognition rate

The main effect of instruction was significant (*F*(1,58) = 22.6, *p* < 0.001, ${{\rm{\eta }}}_{{p}}^{2}=0.280$ ). The words in the remember-cue condition were recognized with a higher rate (83.0 ± 1.3%) than the words in the forget-cue condition (76.5 ± 0.9%).

The main effect of emotion was significant (*F*(1,58) = 5.64, *p* = 0.021, ${{\rm{\eta }}}_{{p}}^{2}=0.089$). Negative words were recognized with a higher rate (80.6 ± 0.9%) compared to neutral words (78.9 ± 1.0%).

**Figure 2:**
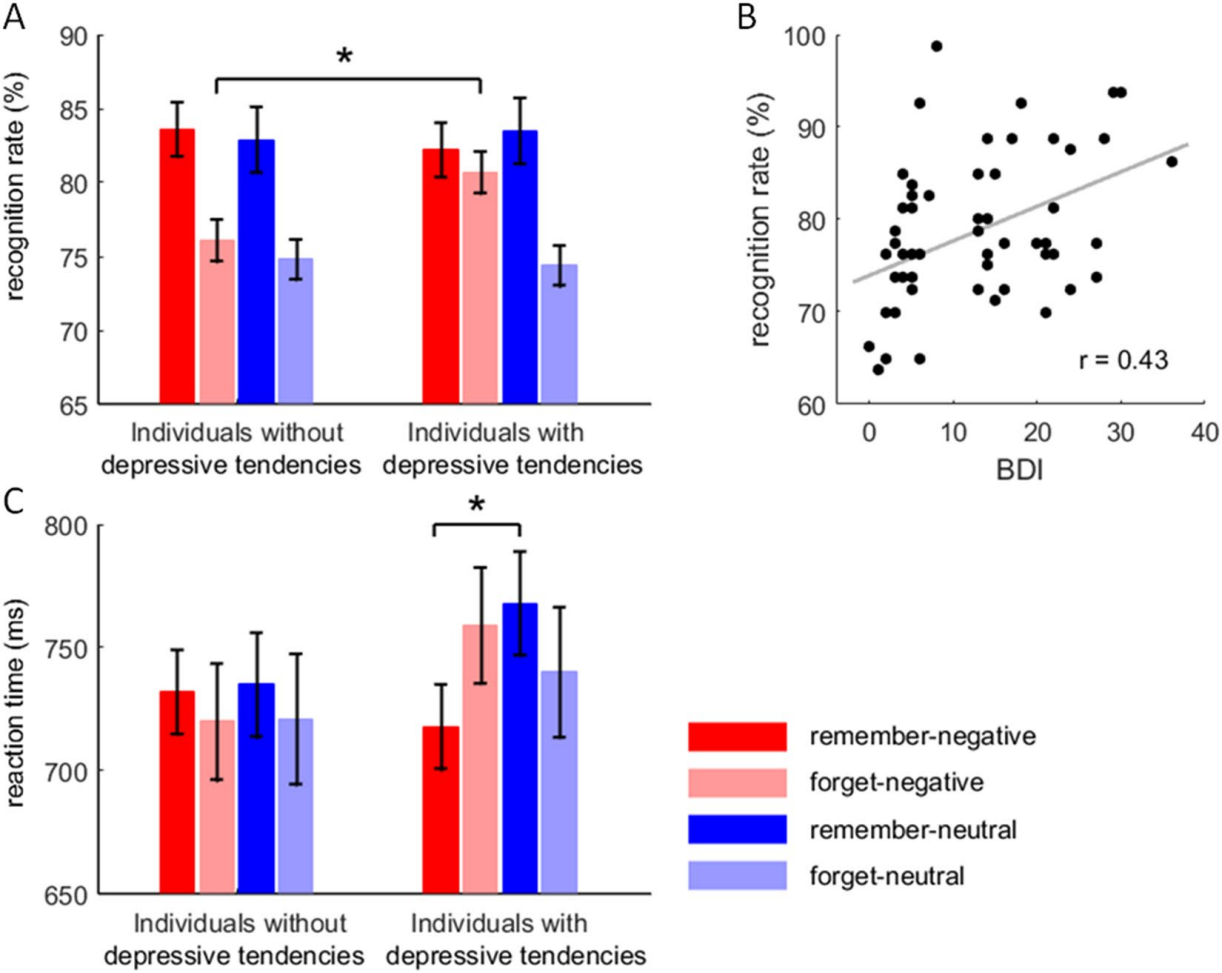
Behavioral results. (**A**) The recognition rate in different conditions. (**B**) Significant correlation between the BDI sore and the recognition rate in the forget-negative-word condition. (**C**) The reaction time in the recognition test. Bars represent ± standard error of the mean.

The interaction of instruction by emotion by group was significant (*F*(1,58) = 6.38, *p* = 0.014, ${{\rm{\eta }}}_{{p}}^{2}=0.099$; Fig. 2A). Compared with subjects without depressive tendencies (76.1 ± 1.4%), subjects with depressive tendencies had a larger recognition rate (80.7 ± 1.4%) when negative words were required to be forgotten (*F*(1,58) = 5.48, *p* = 0.023). However, this group difference did not achieve significant level in any of the other conditions (*F*(1,58) < 1; individuals without depressive tendencies *vs*. with depressive tendencies: remember negative words = 83.6 ± 1.8 *vs*. 82.2 ± 1.8%, remember neutral words = 82.9 ± 2.2 *vs*. 83.5 ± 2.2%, forget neutral words = 74.8 ± 1.3 *vs*. 74.4 ± 1.3%).

#### Reaction time (RT)

The interaction of instruction by emotion by group was significant (*F*(1,58) = 6.58, *p* = 0.013, ${{\rm{\eta }}}_{p\,}^{2}$ = 0.102; Fig. 2C). For the words followed by the remember-cue, subjects with depressive tendencies had a shorter RT when they recognized negative words (718 ± 17.1 ms) than neutral words (768 ± 21.0 ms; *F*(1,58) = 13.6, *p* = 0.001). However, this emotion effect did not achieve significant level in any of the other conditions (*F*(1,58) < 1.42, *p* > 0.238; negative *vs*. neutral: remember in subjects without depressive tendencies = 732 ± 17.1 *vs*. 735 ± 21.0 ms, forget in subjects without depressive tendencies = 720 ± 23.5 *vs*. 721 ± 26.4 ms, forget in subjects with depressive tendencies = 759 ± 23.5 *vs*. 740 ± 26.4 ms).

### ERPs

#### *Word-occipital P1*

No significant effect was found between conditions.

#### Word-frontal P2

The main effect of emotion was significant (*F*(1,58) = 7.52, *p* = 0.008, ${{\rm{\eta }}}_{p\,}^{2}$ = 0.115). Negative words evoked larger P2 amplitudes (5.13 ± 0.46 μV) than neutral words (4.21 ± 0.45 μV) did.

**Figure 3:**
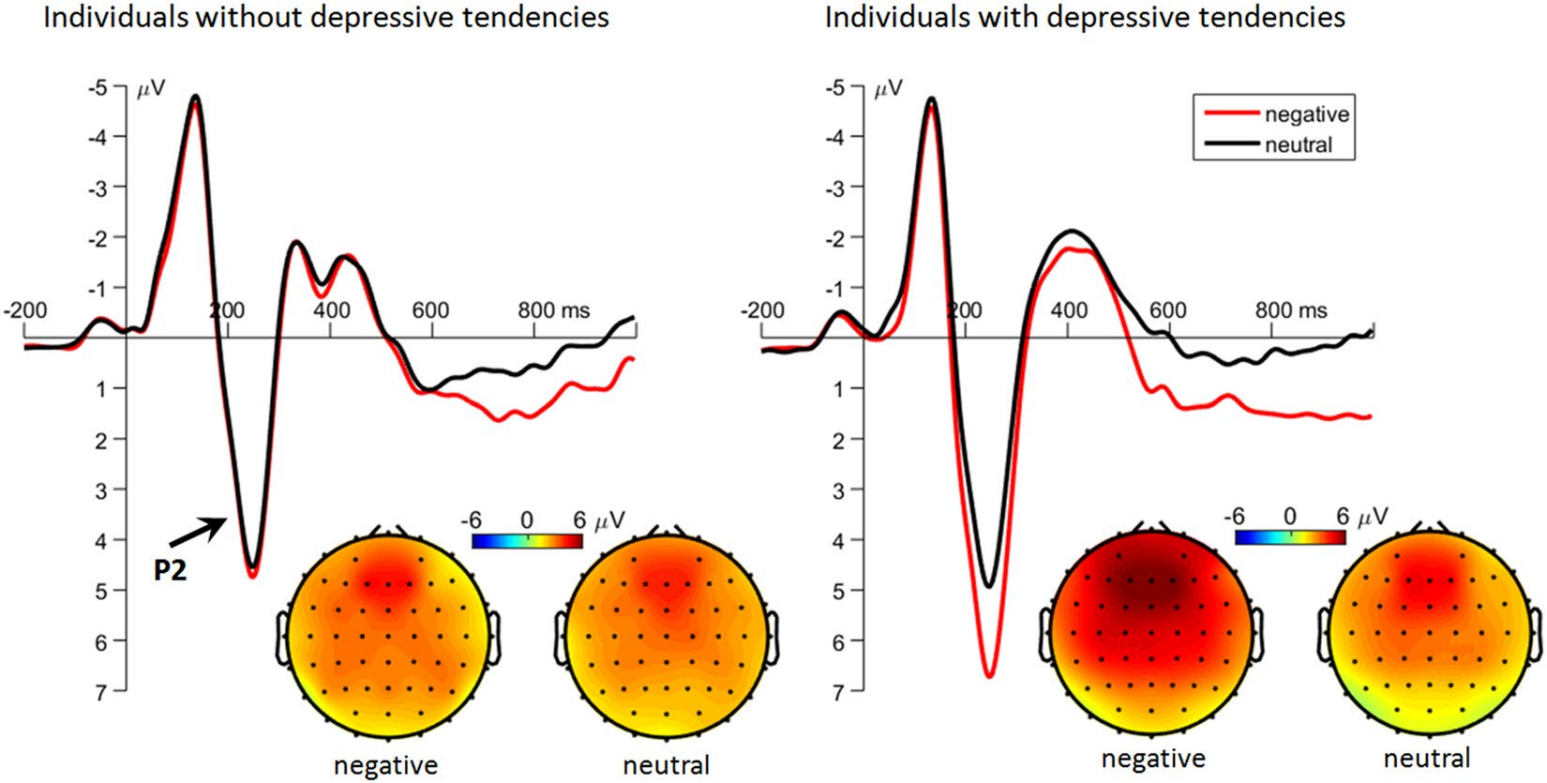
The grand-mean ERP waveforms and topographies of the word-evoked frontal P2 component. Waveforms were calculated by averaging the data at the electrodes of Fz, F1 and F2. Topographies were calculated by averaging the data within a time window of 220 to 270 ms after the onset of words.

The interaction of emotion by group was significant (*F*(1,58) = 5.08, *p* = 0.028, ${{\rm{\eta }}}_{p\,}^{2}$ = 0.081; Fig. 3). Compared with subjects without depressive tendencies (4.09 ± 0.65 μV), subjects with depressive tendencies had larger P2 amplitudes (6.18 ± 0.65 μV) for negative words (*F*(1,58) = 5.14, *p* = 0.027). However, this group difference did not achieve significant level for neutral words (*F*(1,58) < 1; subjects without depressive tendencies = 3.92 ± 0.64 μV, subjects with depressive tendencies = 4.50 ± 0.64 μV).

#### *Word-parietal LPP*

The main effect of emotion was significant (*F*(1,58) = 44.4, *p* < 0.001, ${{\rm{\eta }}}_{p\,}^{2}$ = 0.434). Negative words evoked larger LPP amplitudes (2.35 ± 0.16 μV) than neutral words (1.31 ± 0.17 μV) did.

**Figure 4:**
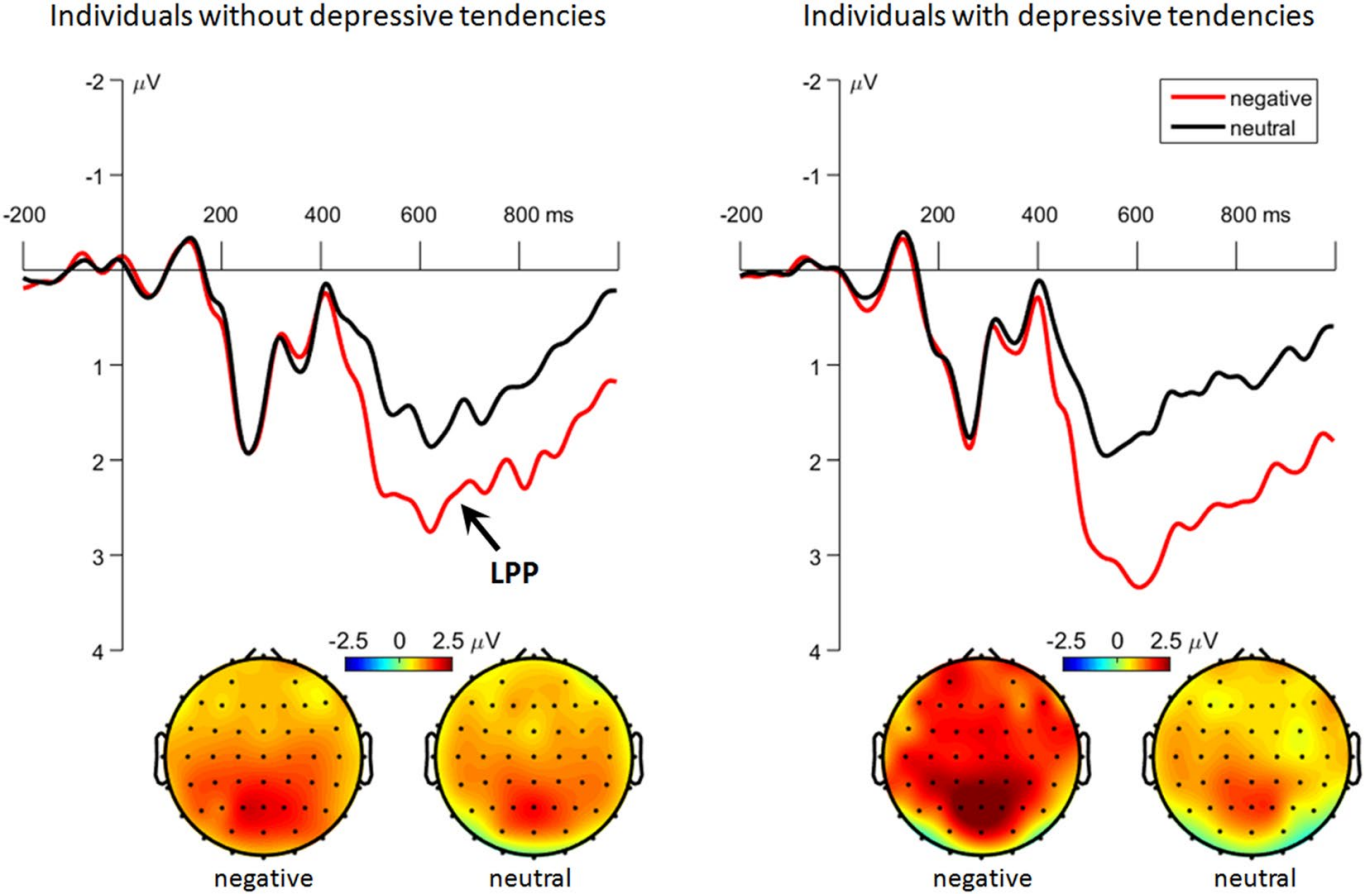
The grand-mean ERP waveforms and topographies of the word-evoked parietal LPP component. Waveforms were calculated by averaging the data at the electrodes of Pz, P1 and P2. Topographies were calculated by averaging the data within a time window of 500 to 900 ms after the onset of words.

The interaction of emotion by group was significant (*F*(1,58) = 4.61, *p* = 0.036, ${{\rm{\eta }}}_{p\,}^{2}$ = 0.074; Fig. 4). Compared with subjects without depressive tendencies (2.01 ± 0.23 μV), subjects with depressive tendencies had larger LPP amplitudes (2.68 ± 0.23 μV) for negative words (*F*(1,58) = 4.16, *p* = 0.046). However, this group difference did not achieve significant level for neutral words (*F*(1,58) < 1; subjects without depressive tendencies = 1.31 ± 0.25 μV, subjects with depressive tendencies = 1.31 ± 0.25 μV).

#### Cue-occipital P1

The main effect of instruction was significant (*F*(1,58) = 44.4, *p* < 0.001, $\,{\eta }_{{\rm{p}}}^{2}\,=$ 0.434). The forget-cue evoked larger P1 amplitudes (1.38 ± 0.14 μV) than the remember-cue (0.44 ± 0.14 μV) did.

The main effect of emotion was significant (*F*(1,58) = 11.5, *p* = 0.001, ${{\rm{\eta }}}_{p\,}^{2}$= 0.165). The P1 amplitude was larger in negative-word trials (1.16 ± 0.14 μV) than in neutral-word trials (0.66 ± 0.14 μV).

**Figure 5:**
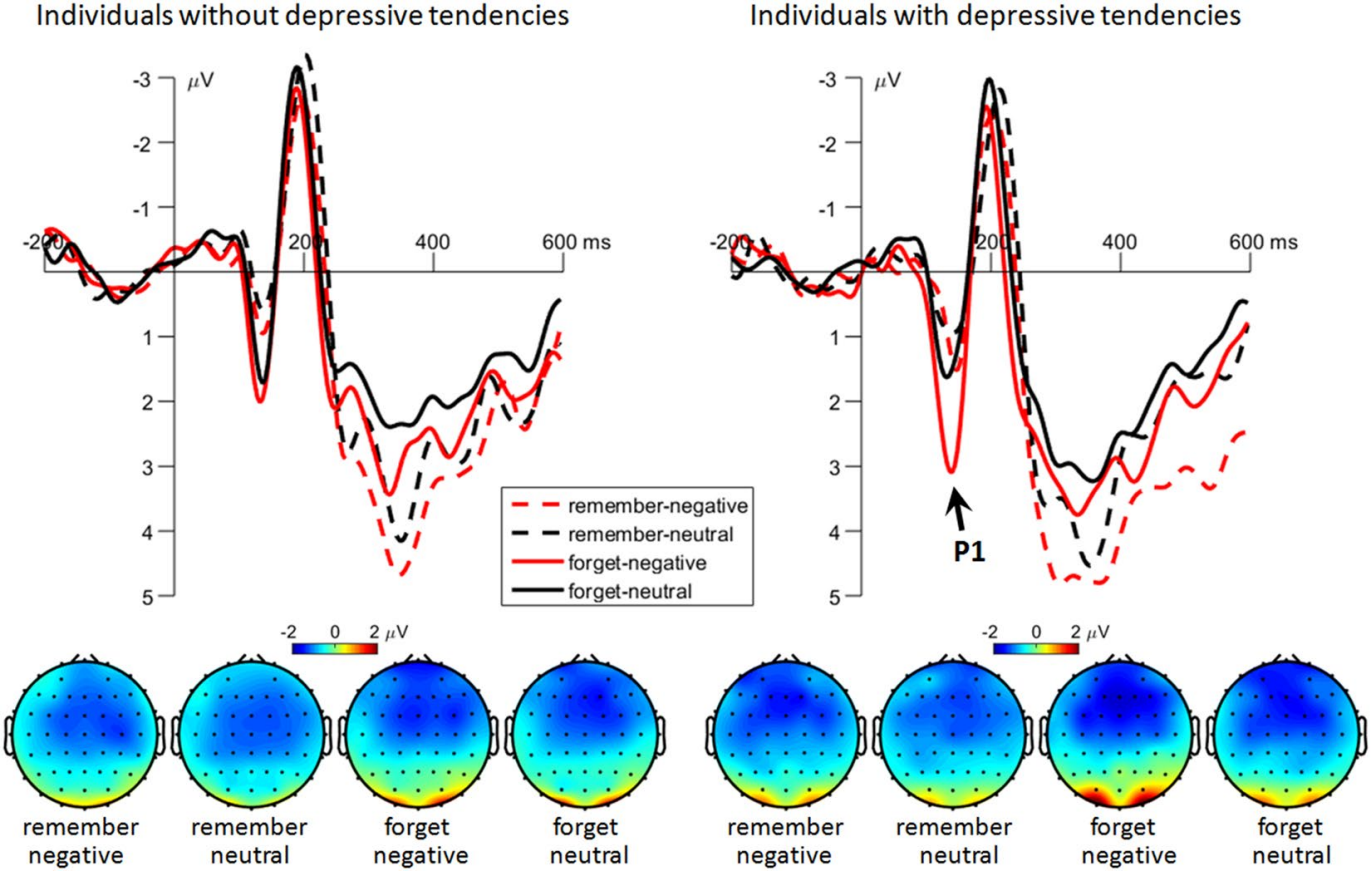
The grand-mean ERP waveforms and topographies of the cue-evoked occipital P1 component. Waveforms were calculated by averaging the data at the electrodes of O1 and O2. Topographies were calculated by averaging the data within a time window of 115 to 155 ms after the onset of cues.

The interaction of instruction by emotion by group was significant (*F*(1,58) = 6.01, *p* = 0.017, ${{\rm{\eta }}}_{p\,}^{2}$ = 0.094; Fig. 5). Compared with subjects without depressive tendencies (1.21 ± 0.24 μV), subjects with depressive tendencies had larger P1 amplitudes (2.30 ± 0.24 μV) in the condition of forgetting previously presented negative words (*F*(1,58) = 10.2, *p* = 0.002). However, this group difference did not achieve significant level in any of the other conditions (*F*(1,58) = 0.55 to 1.74, *p* = 0.192 to 0.461; individuals without *vs*. with depressive tendencies: remember previous negative words = 0.32 ± 0.26 *vs*. 0.80 ± 0.26 μV, remember previous neutral words = 0.12 ± 0.23 *vs*. 0.51 ± 0.23 μV, forget previous neutral words = 1.14 ± 0.23 *vs*. 0.89 ± 0.23 μV).

#### Cue-frontalN2

The main effect of instruction was significant (*F*(1,58) = 43.8, *p* < 0.001, ${\,{\rm{\eta }}}_{{p}}^{2\,}=$ 0.430). The forget-cue evoked larger negative-going amplitudes (2.60 ± 0.17 μV) than the remember-cue (3.78 ± 0.17 μV) did.

The main effect of emotion was significant (*F*(1,58) = 10.1, *p* = 0.002, ${\,{\rm{\eta }}}_{{p}}^{2\,}=$ 0.148). The N2 amplitude was more negative in negative-word trials (2.97 ± 0.17 μV) than in neutral-word trials (3.41 ± 0.16 μV).

**Figure 6:**
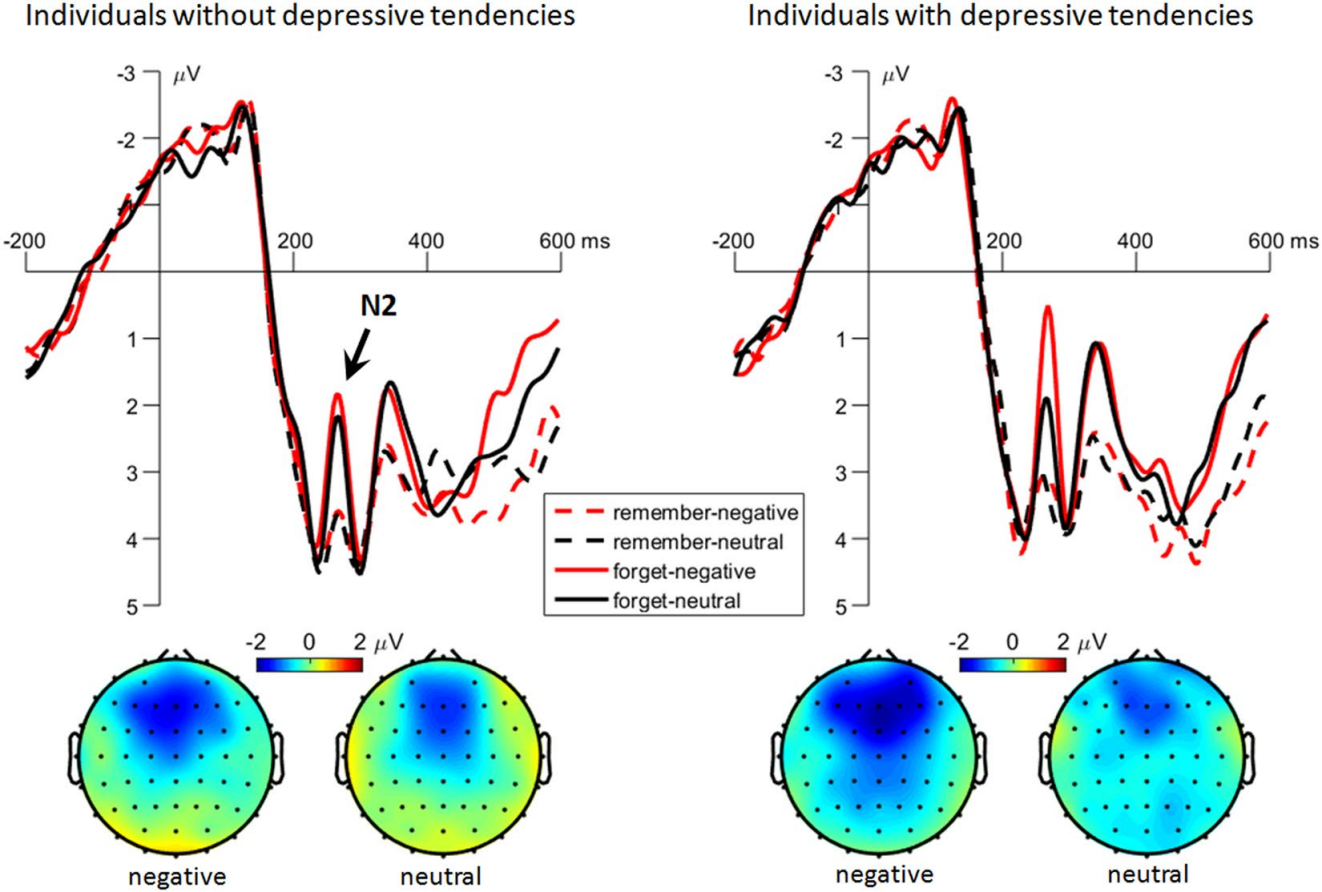
The grand-mean ERP waveforms and topographies of the cue-evoked frontal N2 component. Waveforms were calculated by averaging the data at the electrodes of Fz, F1 and F2. Topographies were calculated by averaging the data within a time window of 240 to 290 ms after the onset of cues. To display negative-going amplitudes for the N2, the topographies were based on the difference amplitudes between forget and remember conditions.

The interaction of instruction by emotion by group was significant (*F*(1,58) = 4.44, *p* = 0.039, ${\,{\rm{\eta }}}_{{p}}^{2\,}=$ 0.071; Fig. 6). Compared with subjects without depressive tendencies (2.90 ± 0.27 μV), subjects with depressive tendencies had larger negative-going N2 amplitudes (1.41 ± 0.27 μV) in the condition of forgetting previous negative words (*F*(1,58) = 15.4, *p* < 0.001). However, this group difference did not achieve significant level in any of the other conditions (*F*(1,58) < 1; individuals without *vs*. with depressive tendencies: remember previous negative words = 3.92 ± 0.29 *vs*. 3.66 ± 0.29 μV, remember previous neutral words = 3.92 ± 0.27 *vs*. 3.64 ± 0.27 μV, forget previous neutral words = 3.18 ± 0.30 *vs*. 2.91 ± 0.30 μV).

#### Cue-parietal P3

**Figure 7:**
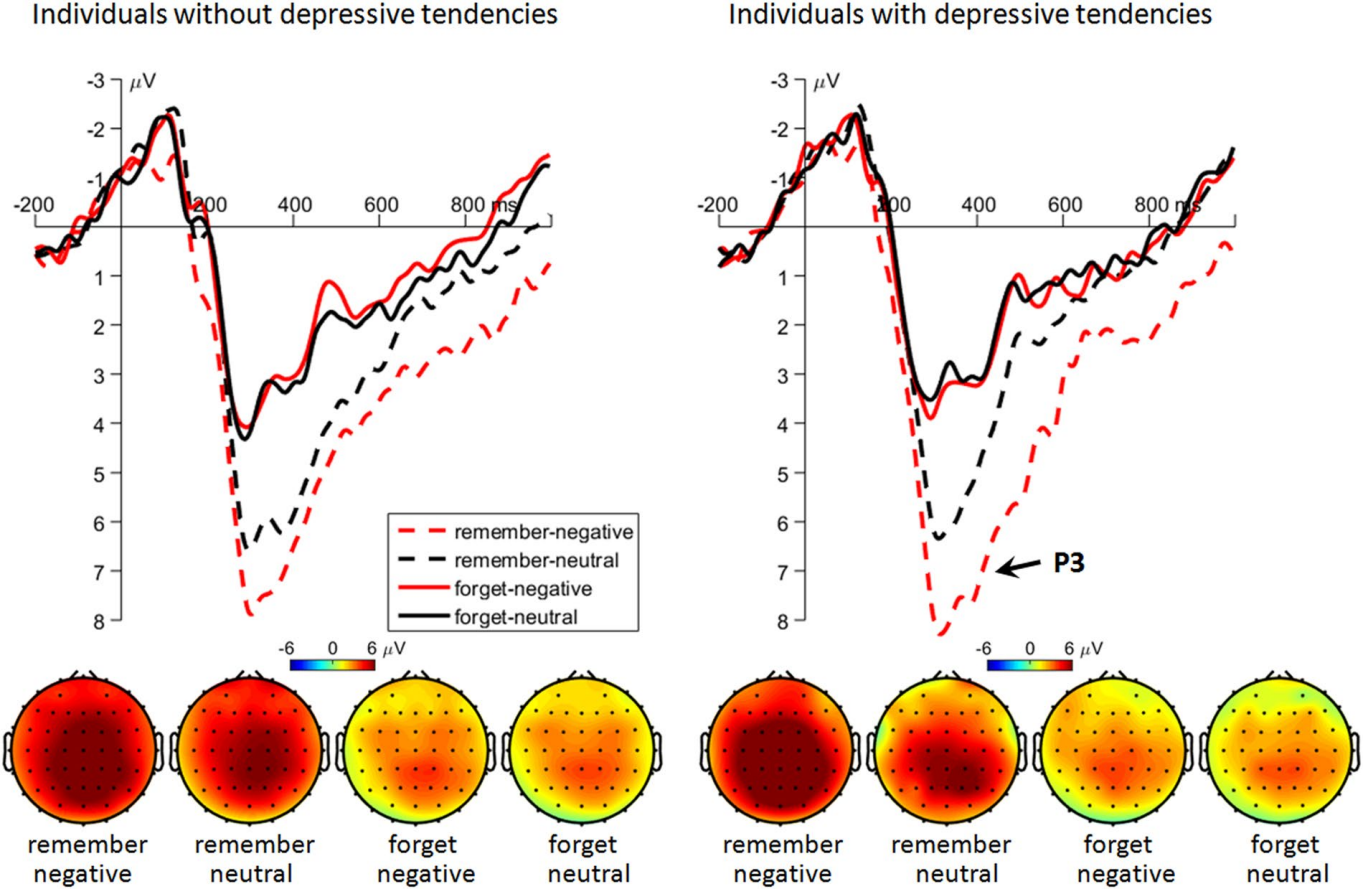
The grand-mean ERP waveforms and topographies of the cue-evoked parietal P3 component. Waveforms were calculated by averaging the data at the electrodes of CPz, CP1 and CP2. Topographies were calculated by averaging the data within a time window of 260 to 460 ms after the onset of cues.

The main effect of instruction was significant (*F*(1,58) = 198, *p* < 0.001, ${\,{\rm{\eta }}}_{{p}}^{2\,}=$ 0.774; Fig. 7). The remember-cue evoked larger P3 amplitudes (5.96 ± 0.21 μV) than the forget-cue (3.19 ± 0.21 μV) did.

The main effect of emotion was significant (*F*(1,58) = 19.4, *p* < 0.001, ${\,{\rm{\eta }}}_{{p}}^{2\,}=$ 0.251). The P3 amplitude was larger in negative-word trials (4.99 ± 0.20 μV) than in neutral-word trials (4.16 ± 0.22 μV).

### Correlation

According to the results reported above, correlations were performed between the five self-reported scores (BDI, STAI-T, RRS, BIS and BAS) and the six behavioral/ERP indices (i.e., the recognition rate in the forget-negative-word condition, the RT in the remember-negative-word condition, the word-evoked P2 and LPP in the negative condition, and the cue-evoked P1 and N2 in the forget-negative-word condition). In total, we performed 30 (5 × 6) correlations.

**Figure 8:**
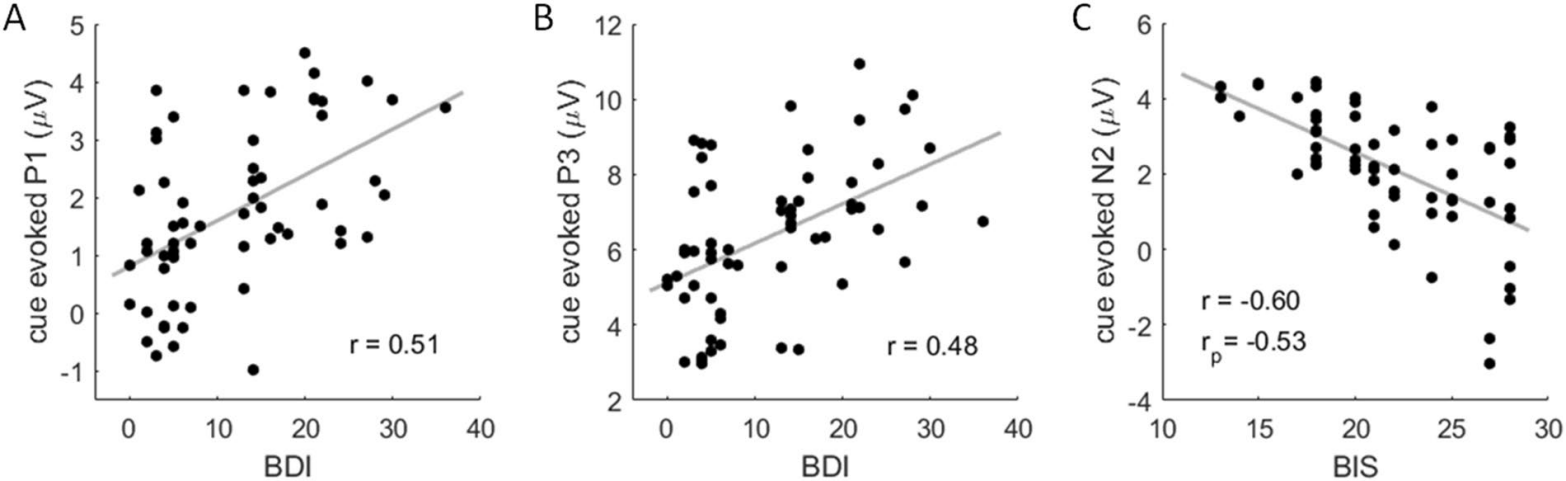
Significant correlations between self-reported measures and ERP data. (**A**) Correlation between the BDI sore and the cue-evoked P1 amplitude in the forget-negative-word condition. (**B**) Correlation between the BDI sore and the cue-evoked P3 amplitude in the remember-negative-word condition. (**C**) Correlation between the BIS sore and the cue-evoked N2 amplitude in the forget-negative-word condition. Note that the N2 is a negative-going component, so the correlation reveals that larger BIS scores were correlated with larger N2 amplitudes. (**D**) Correlation between the RRS sore and the cue-evoked P3 amplitude in the remember-negative-word condition.

Results showed four significant correlations after correction for multiple comparisons. The BDI score was correlated with the recognition rate (*r* = 0.431, *p* < 0.001, corrected *p* = 0.016; Fig. 2B), the amplitudes of cue-evoked P1 (*r* = 0.512, *p* < 0.001, corrected *p* < 0.001), and cue-evoked P3 (*r* = 0.485, *p* < 0.001, corrected *p* = 0.002) (Fig. 8A and B). The BIS score was correlated with the amplitude of cue-evoked N2 (*r* = −0.604, *p* < 0.001, corrected *p* < 0.001) (Fig. 8C) (Note: Since the N2 is a negative-going component, the negative correlation means that higher BIS scores were associated with larger N2 amplitudes).

After controlling for the other four self-reported measures, only one significant correlation was detected. The BIS score was correlated with the amplitude of cue-evoked N2 (*r*_p_ = −0.527, *p* < 0.001, corrected *p* < 0.001).

In addition, it was found that the three self-reported measures were pairwise correlated (BDI and RRS: *r* = 0.808, *p* < 0.001; BDI and BIS: *r* = 0.539, *p* < 0.001; BIS and RRS: *r* = 0.580, *p* < 0.001).

## Discussion

In the current study, the ability of intentional forgetting of negative and neutral materials in individuals with depressive tendencies was examiend. It was demonstrated that this population had difficulties in suppressing the memory encoding of negative items, while the suppression of memory encoding of neutral words was relatively intact. The behavioral results suggest that the deficit of memory inhibition is specifically pronounced for negative stimuli, which is consistent with previous studies^8,9,31,32^.

The ERP data revealed abnormal word-evoked (P2 and LPP) and cue-evoked (P1 and N2) components for negative words in participants with depressive tendencies. In contrast to the hypothesis, negative words did not elicit larger cue-evoked P3 amplitudes for TBR items in subjects with depressive tendencies, compared to the control group. However, this result was consistent with the obtained recognition rate (subjects with depressive tendencies did not remember more negative words than nondepressed subjects), and other studies^9,31,32^.

Based on these results, we propose that two mechanisms may contribute to the failure of forgetting negative material in individuals with depressive tendencies.

### Mechanism 1: Inefficient selective attention to and suppression of negative material

The most important finding of this study was the N2 interactive effect; the “forget” instructive cue elicited larger N2 for negative words in subjects with, compared to subjects without, depressive tendencies. The frontal N2 is generally identified as an ERP marker of inhibitory control^19,23,37,68^. Accordingly, the “forget” cue evoked an obvious N2 while the “remember” cue did not. The data also revealed a main effect of emotion; larger N2 amplitudes were associated with negative words than with neutral words. This emotional effect is consistent with previous DF findings in healthy people, demonstrating that individuals usually recruit more inhibitory resources (indicated by larger N2 amplitudes) to suppress negative memories^23^ because forgetting negative stimuli is more laborious and less efficient^16,20,33,69^. In line with this interpretation, the N2 interaction of the current study suggests that subjects with depressive tendencies recruit even more inhibitory control resources to reduce the long-term memory of negative stimuli than nondepressed subjects, but this compensatory effort does not make up for their inefficiency in intentional forgetting. This is consistent with what was reported by Yang *et al*., who found that forgetting negative items, compared to neutral items, caused enhanced activation in the inferior and superior frontal gyrus in depressed patients^9^. Furthermore, the proposed association between the “compensatory effort” and the enhanced N2 was confirmed by the correlation between the BIS score and the N2 amplitude: a larger N2 in the forget-negative-word condition was associated with a larger motivation (or effort) to keep away from negative stimuli. This correlation was observed in both depressed and nondepressed individuals.

Another interesting finding was that the cue-evoked P1 displayed exactly the same pattern as the cue-evoked N2: the instruction of forgetting negative words evoked larger P1 amplitudes in subjects with depressive tendencies, than that in subjects without depressive tendencies. The occipital P1 is an early ERP component associated with feature-based selective attention^45^ and it is sensitive to task demands^70^ and arousal levels^46^. The P1 result indicates that the “forget” cue following negative words attracted more early attention in individuals with depressive tendencies, compared to individuals without depressive tendencies. It is possible that the enhanced P1 in depressed individuals reflected a heightened unconscious amplification of the instruction of “forget the previous negative word” so as to facilitate the task^71,72^. Alternatively, the “forget-negative-word” cue evoked a higher level of arousal in subjects with depressive tendencies. Similar with this finding, previous studies demonstrated that depressed subjects have larger P1 amplitudes compared with nondepressed subjects, especially following the presentation of negative material^73–77^. However, since this is the first report of this P1 finding in the DF literature, further study is needed to verify the speculation.

Altogether, individuals with depressive tendencies, on the one hand, could not forget negative words successfully, and on the other hand, allocated more cognitive resources than nondepressed ones when suppressing negative material. Therefore, the present study proposes that the selective attention system (P1) and the inhibitory control system (N2) function in an inefficient way in individuals with depressive tendencies (or mild depression).

### Mechanism 2: Excessive preliminary processing of negative material

Beyond what the previous studies reported on the abnormal neural activation in the implementation of the remember/forget cue^8,9^, this study also provided evidence for the excessive preliminary processing of negative words in individuals with depressive tendencies: during the encoding procedure, negative words elicited larger P2 and LPP in subjects with depressive tendencies, compared to subjects without depressive tendencies.

The discovery of this LPP interactive effect is in accordance with the hypothesis of this study. The LPP has been widely considered to reflect sustained attentional allocation and continuous processing of emotional stimuli^73,78–82^. The enhanced LPP for negative compared to neutral items suggests that negative items were processed more extensively than neutral items, which is consistent with previous DF studies^20,23,33,34^. In line with this interpretation, the even larger LPP for negative words in subjects with depressive tendencies indicates a more intensive encoding of negative material.

An unexpected finding was that the frontal P2 was larger in response to negative than to neutral words in participants with depressive tendencies. According to previous literature, P2 is associated with the motivational salience of a stimulus and usually reflects motivation-related attentional allocation^48–50^. Many emotional studies found larger P2 amplitudes in response to negative than to neutral and positive stimuli^83–87^, indicating an attentional bias towards negative material. Furthermore, Bernat *et al*. proposed that the P2 plays a key role in determining which emotional stimuli become conscious ones, since the time window of 200 to 300 ms is on the boundary of nonconscious and conscious processing^88^. In this study, nondepressed subjects did not display differential P2 amplitudes between negative and neutral conditions, while subjects with depressive tendencies elicited larger P2 when viewing negative words. This result suggests that negative items may have more motivational significance to individuals with depressive tendencies, who exhibit excessive negative biases in many aspects of the cognitive processes^1,2^.

Taken together, the enhanced word-evoked P2 and LPP indicate an excessive preliminary processing of negative items in individuals with depressive tendencies, which may result in the subsequent failure of directed forgetting of negative material^20,23^. Although this excessive processing of negative words did not lead to higher recognition rate for negative TBR items in these individuals, a significantly shorter reaction time was observed in this group for negative compared to neutral TBR items.

### Nondepressed individuals are able to suppress negative material

This study also found that, nondepressed participants could successfully forget both neutral and negative materials.

Previous DF studies in healthy individuals resulted in conflicting conclusions on the issue of emotional bias. Some proposed that participants failed to forget negative material^20,33,69^, while others argued that both neutral and negative material could be intentionally forgotten^16,23,34^. The inhomogeneity of experimental samples may be one of the reasons for the inconsistent results. After controlling for individual differences such as depression and trait anxiety, this study provided evidences which support the latter argument. Also, it was indicated that the BDI score was positively correlated with the recognition rate of negative TBF items. This result prompts the consideration of the level of depression as a variable when examining the emotional DF effect in a given population.

### Limitations

Two limitations should be considered for an appropriate interpretation of the current result. First, two possible mechanisms that may contribute to the failure of forgetting negative material in individuals with depressive tendencies were proposed. However, since the results indicated that the individuals with depressive tendencies also excessively processed negative material (reflected by enhanced word-evoked P2 and LPP), the recruitment of additional inhibitory resources in the implementation of the forget cue (reflected by enhanced cue-evoked N2) may be a compensation mechanism for this excessive processing, rather than due to the inefficiency of memory suppression of negative material. Thus, further studies using other paradigms are required to clarify this issue. Second, this study considered undergraduate students who scored > 13 in BDI-II as individuals with depressive tendencies. Thus the participants in the depressive tendency group were mildly depressed individuals. Further study is necessary to test the generalizability of the current conclusion to clinically depressed patients. Furthermore, because anxiety and depressive symptoms are highly comorbid, this study only selected participants with a moderate level of trait anxiety.

## Conclusion

The current study showed that individuals with depressive tendencies experienced difficulty in forgetting negative material. Individuals with depressive tendencies displayed enhanced word-evoked P2 and LPP for negative items and enhanced cue-evoked P1 and N2 for negative TBF items, compared to individuals without depressive tendencies. Consequently, we proposed two mechanisms during memory encoding that may contribute to the failure to forget negative material in mild depression. The two proposed mechanisms, which may influence the intentional forgetting in a negatively valenced context, are the inefficient early selective attention and memory suppression (reflected by cue-evoked P1 and N2) and the excessive preliminary processing (reflected by word-evoked P2 and LPP).

## Electronic supplementary material

Supplementary material
