# Supplementary material for: Individuals with depressive tendencies experience difficulty in forgetting negative material: two mechanisms revealed by ERP data in the directed forgetting paradigm
Source: Sci Rep. 2018 Jan 18;8:1113. doi: 10.1038/s41598-018-19570-0 (PMC5773552; doi:10.1038/s41598-018-19570-0)
Supplement: Supplementary file 1 — Supplementary material [file 41598_2018_19570_MOESM1_ESM.pdf]

Individuals with depressive tendencies experience difficulty in forgetting negative material: two mechanisms revealed by ERP data in the directed forgetting paradigm

Hui Xie[#], Donghong Jiang[#], Dandan Zhang*

Institute of Affective and Social Neuroscience, Shenzhen University, Shenzhen 518060, China

Shenzhen Key Laboratory of Affective and Social Cognitive Science，Shenzhen University, Shenzhen 518060, China

* Corresponding author. Room 412, Building of Normal School, Shenzhen University, 3688 Nanhai Ave, Nanshan District, Shenzhen 518060, P.R. China . Tel/Fax：86-755-26421385. E-mail: zhangdd05@gmail.com

# Both authors contributed equally to the study.

Running title: Forgetting negative material in depression

Additional results

Behavioral data

Recognition rate

The interaction of instruction by emotion was significant ($F(1,58) = 8.65$, $p = 0.005$, $\eta_p^2 = 0.130$). Compared with the instruction of forgetting neutral words (74.6 ± 1.0 %), the instruction of forgetting negative words resulted a larger recognition rate (78.4 ± 1.0 %; $F(1,58) = 19.2$, $p < 0.001$). However, this emotion difference did not achieve significant level when participants were instructed to remember previously presented words ($F(1,58) < 1$; negative = 82.9 ± 1.3 %, neutral = 83.2 ± 1.6 %).

Reaction time

The interaction of instruction by emotion was significant ($F(1,58) = 7.10$, $p = 0.010$, $\eta_p^2 = 0.109$). Compared with the condition of remembering neutral words (751 ± 14.8 ms), the condition of remembering negative words was associated with a shorter RT (725 ± 12.1 ms) ($F(1,58) = 7.39$, $p = 0.009$). However, the emotion difference did not achieve significant level when participants were instructed to forget previously presented words ($F(1,58) < 1$; negative = 739 ± 16.6 ms, neutral = 731 ± 18.6 ms).

ERPs

Cue-occipital P1

The interaction of emotion by group was significant ($F(1,58) = 5.96$, $p = 0.018$, $\eta_p^2 = 0.093$). Compared with subjects without depressive tendencies (0.76 ± 0.20 μV), subjects with depressive tendencies had larger P1 amplitudes (1.55 ± 0.20 μV) in negative-word trials ($F(1,58) = 7.75$, $p = 0.007$). However, this group difference did not achieve significant level in neutral-word trials ($F(1,58) < 1$; nondepressed = 0.63 ± 0.20 μV, depressed = 0.70 ± 0.20 μV).

Cue-frontal N2

The interaction of emotion by group was significant ($F(1,58) = 4.73$, $p = 0.034$, $\eta_p^2 = 0.075$). Compared with subjects without depressive tendencies (3.41 ± 0.24 μV), subjects with depressive tendencies had larger negative-going N2 amplitudes (2.53 ± 0.24 μV) in negative-word trials ($F(1,58) = 7.00$, $p = 0.010$). However, this group difference did not achieve significant level in neutral-word trials ($F(1,58) < 1$; nondepressed = 3.55 ± 0.22 μV, depressed = 3.27 ± 0.22 μV).

The interaction of instruction by emotion was significant ($F(1,58) = 9.61$, $p = 0.003$, $\eta_p^2 = 0.142$). Compared with the instruction of forgetting previous neutral words (3.05 ± 0.21 μV), the instruction of forgetting previous negative words evoked larger negative-going N2 amplitudes (2.15 ± 0.19 μV; $F(1,58) = 18.8$, $p < 0.001$). However, this emotion difference did not achieve significant level when participants were instructed to remember previously presented words ($F(1,58) < 1$; negative = 3.79 ± 0.20 μV, neutral = 3.78 ± 0.19 μV).

Cue-parietal P3

The interaction of instruction by emotion was significant ($F(1,58) = 23.7$, $p < 0.001$, $\eta_p^2 = 0.290$). Compared with the instruction of remembering neutral words (5.16 ± 0.26 μV), the instruction of remembering negative words evoked larger P3 amplitudes (6.77 ± 0.24 μV; $F(1,58) = 43.1$, $p < 0.001$). However, this emotion difference did not achieve significant level when participants were instructed to forget previously presented words ($F(1,58) < 1$; negative = 3.22 ± 0.22 μV, neutral = 3.16 ± 0.27 μV).
